# Quantification of Local Morphodynamics and Local GTPase Activity by Edge Evolution Tracking

**DOI:** 10.1371/journal.pcbi.1000223

**Published:** 2008-11-14

**Authors:** Yuki Tsukada, Kazuhiro Aoki, Takeshi Nakamura, Yuichi Sakumura, Michiyuki Matsuda, Shin Ishii

**Affiliations:** 1 Laboratory for Systems Biology, Graduate School of Information Science, Nara Institute of Science and Technology, Nara, Japan; 2 Institute for Bioinformatics Research and Development, Japan Science and Technology Agency, Tokyo, Japan; 3 Laboratory of Bioimaging and Cell Signaling, Graduate School of Biostudies, Kyoto University, Kyoto, Japan; 4 Integrated Systems Biology Laboratory, Department of Systems Science, Graduate School of Informatics, Kyoto University, Kyoto, Japan; Nagoya University, Japan

## Abstract

Advances in time-lapse fluorescence microscopy have enabled us to directly observe dynamic cellular phenomena. Although the techniques themselves have promoted the understanding of dynamic cellular functions, the vast number of images acquired has generated a need for automated processing tools to extract statistical information. A problem underlying the analysis of time-lapse cell images is the lack of rigorous methods to extract morphodynamic properties. Here, we propose an algorithm called edge evolution tracking (EET) to quantify the relationship between local morphological changes and local fluorescence intensities around a cell edge using time-lapse microscopy images. This algorithm enables us to trace the local edge extension and contraction by defining subdivided edges and their corresponding positions in successive frames. Thus, this algorithm enables the investigation of cross-correlations between local morphological changes and local intensity of fluorescent signals by considering the time shifts. By applying EET to fluorescence resonance energy transfer images of the Rho-family GTPases Rac1, Cdc42, and RhoA, we examined the cross-correlation between the local area difference and GTPase activity. The calculated correlations changed with time-shifts as expected, but surprisingly, the peak of the correlation coefficients appeared with a 6–8 min time shift of morphological changes and preceded the Rac1 or Cdc42 activities. Our method enables the quantification of the dynamics of local morphological change and local protein activity and statistical investigation of the relationship between them by considering time shifts in the relationship. Thus, this algorithm extends the value of time-lapse imaging data to better understand dynamics of cellular function.

## Author Summary

Morphological change is a key indicator of various cellular functions such as migration and construction of specific structures. Time-lapse image microscopy permits the visualization of changes in morphology and spatio-temporal protein activity related to dynamic cellular functions. However, an unsolved problem is the development of an automated analytical method to handle the vast amount of associated image data. This article describes a novel approach for analysis of time-lapse microscopy data. We automated the quantification of morphological change and cell edge protein activity and then performed statistical analysis to explore the relationship between local morphological change and spatio-temporal protein activity. Our results reveal that morphological change precedes specific protein activity by 6–8 min, which prompts a new hypothesis for cellular morphodynamics regulated by molecular signaling. Use of our method thus allows for detailed analysis of time-lapse images emphasizing the value of computer-assisted high-throughput analysis for time-lapse microscopy images and statistical analysis of morphological properties.

## Introduction

Cell morphological change is a key process in the development and homeostasis of multicellular organisms [1],[2]. Various types of morphological change appear during migration and differentiation; essential events occurring as part of these processes usually accompany morphologically different phenotypes. Therefore, cell morphology has been used as a key indicator of cell state [3]. High-throughput analyses of cell morphodynamic properties have been used recently to discover new functions of specific proteins [4]. Moreover, the outcomes of morphological change such as the intricate shape of neuronal dendrites, remind us that morphogenesis itself plays a role in the emergence of cellular function [5].

Quantitative approaches are helping to unveil cellular morphodynamic systems, and they are generating new technical requirements. Because cellular morphological change is highly dynamic, time-lapse imaging is necessary to understand the mechanism of cell morphology regulation. Progress in the development of fluorescent probes has enabled the direct observation of cell morphological changes and/or the localization and activity of specific proteins [6]–[8], but time-lapse imaging has highlighted the difficulty of extracting characteristic information from an immense number of images. Nevertheless, several approaches in the context of quantitative analysis have appeared recently. A series of studies using quantitative fluorescent speckle microscopy, for instance, revealed the power of computer-assisted high-throughput analysis for time-lapse microscopy images: analysis of the number of moving and blinking speckles suggested distinct regulation of actin reorganization dynamics in different intracellular regions [9],[10].

Indeed, computational methods have been used to determine the properties of morphological dynamics, protein activity and gene expression [11]–[14]. There are two major approaches for the detailed analysis of local morphological changes of cells. One is the kymograph, which is a widely used method to describe motion with a time-position map of the morphology time course. The time course of change in intensity could also be monitored by arranging sequential images of a specific region of interest (ROI) [15]. Although there are drawbacks to this approach, such as restriction of the analyzed area to a narrow ROI and the need to manually define the ROI, recent studies have avoided these limitations by using polar coordinates to explore the motility dynamics of the entire peripheral region of round cells. Indeed, the polar coordinate-based approach showed isotropic and anisotropic cell expansion, and examined stochastic, transient extension periods (named STEP) or periodic contractions [12],[16]. The second approach is to track cellular edge boundaries by tracing virtually defined markers. Kass and Terzopoulos introduced an active contour model known as SNAKES [17], which is widely used to analyze moving video images in applications including biomedicine. For example, Dormann et al. used SNAKES to quantify cell motility and analyze the specific translocation of PH domain-containing proteins into the leading edge [14]. Marker-based tracking has advantages in quantifying highly motile cell morphology, because it does not require a fixed axis, which is necessary in the kymograph approach. Recently, Machacek and Danuser developed an elegant framework to trace a moving edge, using marker tracking modified by the level set method to elucidate morphodynamic modes of various motile cells such as fibroblasts, epithelial cells, and keratocytes [18].

**Figure 1:**
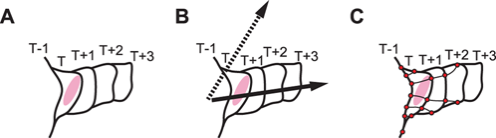
Obstacles to quantifying cell morphological changes. (A) General scheme of cellular morphological changes. The diagram shows part of a cell's edge expanding continuously over time (frame number) *T*−1 to *T*+3. We focus on the correlation timing between morphological change and a regulation signal (red region). (B) The kymograph approaches, including polar coordinate-based analysis, encounters problem caused by the fixed direction of the axis. Although it describes morphodynamics along the proper direction of the axis (solid arrow), lateral movements against this assigned direction (dotted arrow) cannot be quantified. (C) Marker-based analysis rearranges the marker positions depending on the rate and direction of morphological changes, so that the marker density cannot be conserved. Therefore, it is not suitable for persistently changing cell morphology such as neurite outgrowth.

Although previous methodologies have successfully described the specific aspects of cellular morphodynamics, there remain challenges to quantify the relationship between morphodynamics and signaling events. One representative problem is the association between regions in different frames. To scrutinize the dynamic relationship between morphological change and molecular signaling, we need to cross-correlate them in a time-dependent manner (Figure 1A). A polar coordinate system does not ensure the association of time-shifted local domains (Figure 1B), and is unsuitable for non-circular cell shapes. The virtual marker tracking method satisfies this requirement for cells with broadly consistent shapes, but its fixed number of markers causes unequal distribution when a dramatic shape change such as the persistent growth of neurites in neurons, occurs (Figure 1C). Taking these problems into account, we perceive the need for a novel quantification method to better understand the mechanisms of morphodynamic regulation by molecular signaling.

**Figure 2:**
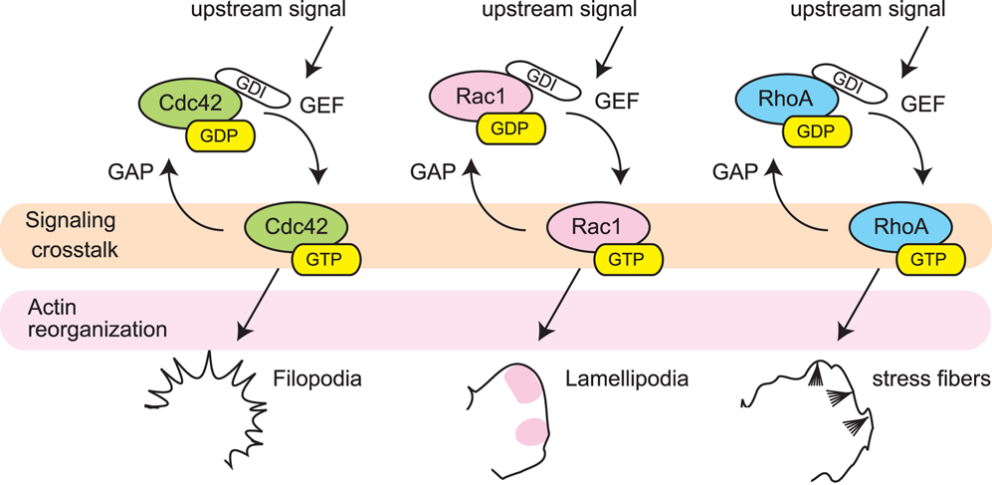
GTPase cascades involved in morphological regulation and cytoskeleton organization. Various upstream signals trigger the activation of Cdc42, Rac, and Rho GTPases and induce morphological and cytoskeletal changes such as formation of filopodia, lamellipodia, and stress fibers, respectively. The ratio of the inactive GDP-bound state to active GTP-bound state is regulated by guanine nucleotide exchange factors (GEFs) and the GTPase-activating proteins (GAPs). Many studies have shown crosstalk between these GTPases; however, direct links between these GTPases are still to be clarified.

We focused on the Rho-family small GTPases, or Rho GTPases, as signaling molecules associated with cell morphodynamics. Rho GTPases, which act as binary switches by cycling between inactive and active states (Figure 2), play key roles in linking biochemical signaling with biophysical cellular behaviors [19],[20] mainly through reorganization of the actin and microtubule cytoskeleton [21]. It is well known that RhoA, Rac1, and Cdc42 have unique abilities to induce specific filamentous actin structures, i.e., stress fibers, lamellipodia, and filopodia, respectively [19]. Considerable evidence, mainly obtained using constitutively-active or dominant-negative mutants, supports a promotional role of Rac1 and Cdc42 and an inhibitory role of RhoA in cell protrusion [19],[21]. Although some researchers have challenged this widely-accepted notion in a variety of cell contexts [22]–[24], our current study has been motivated by this predominant view.

The objective of this study was to uncover the relationship between spatio-temporal activities of Rho GTPases and morphological changes of the cells. To achieve this, we needed a data analysis tool to assess the link between biochemical signaling and biophysical phenomena. However, we do not focus on unveiling the orchestration of the complete signaling pathways that regulate cell morphology. In addition, we elucidated how Rho GTPases regulate “two-dimensional” morphological changes of cells, rather than “three-dimensional” changes. These findings will however be meaningful because the results can be compared with earlier findings [25]–[28]. Therefore, we first present an algorithm called edge evolution tracking (EET) to quantify local morphological change. The main features of our method are that (1) identification of a local morphological change is based on an area difference between two consecutive frames; (2) cell edge is not characterized by point markers, but by line segments, which are defined by the area difference; and (3) past history and future evolution of each segment can be evaluated by connecting segments between consecutive frames. Therefore, this method enables us to trace complex cell edge extension and contraction while maintaining the consistency of the ROI during the analysis. Second, applying EET to fluorescence resonance energy transfer (FRET) time-lapse images of three Rho GTPases (Rac1/Cdc42/RhoA), we found a significant time-shifted cross-correlation between morphological change and GTPase activity. Our study reveals the utility of detailed cellular morphodynamic profiling and spatio-temporal signal profiling to measure the time-shifted relationship between morphodynamics and protein activity.

## Materials and Methods

### Edge Evolution Tracking

**Figure 3:**
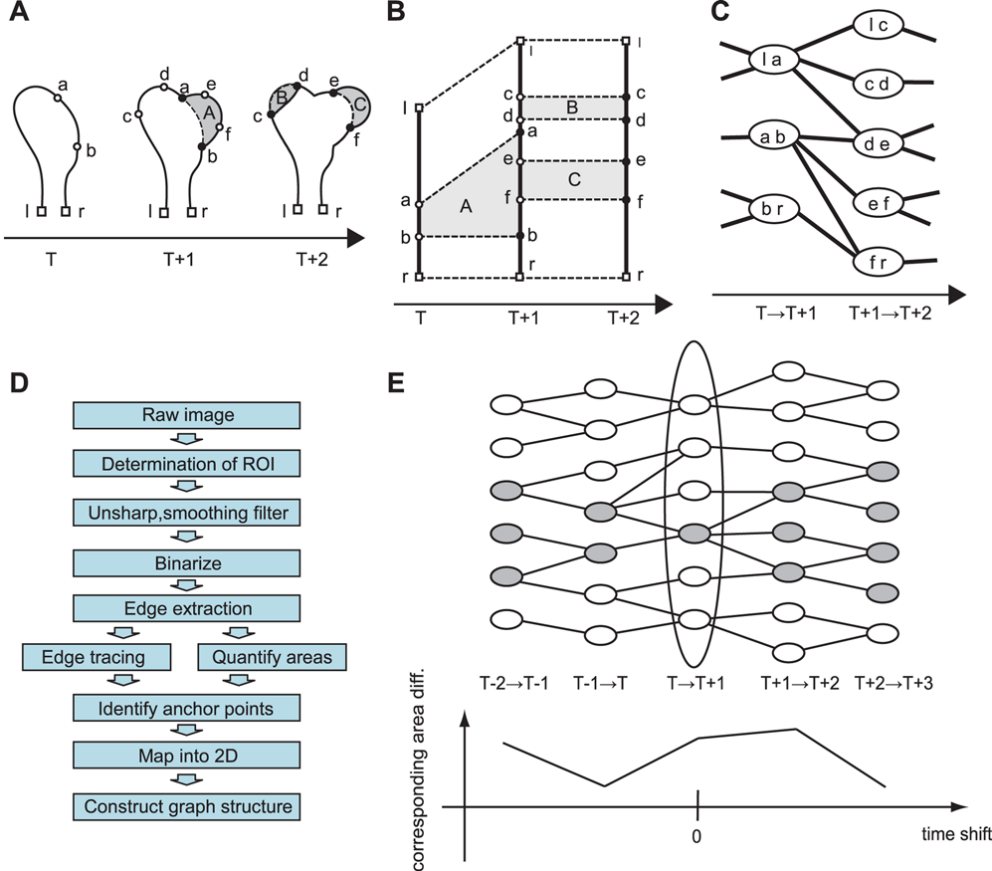
Schematic view of edge evolution tracking. (A) Identification of morphodynamic properties. Solid lines denote cellular edge at each frame and the shaded regions A, B, and C indicate area differences between consecutive frames. We define two properties for a local morphological status transition: segments and anchor points. The segments are subdivided along the cellular edges, which are determined by the area differences between neighboring frames. The anchor points are segment terminals (closed circles) and are projected into the previous frame (open circles). Open squares l and r represent the edge terminals. (B) All of the segments identified and anchor points are mapped two-dimensionally. Horizontal and vertical axes denote the time and position along the cell edge, respectively. Connections between anchor points (dashed lines) illustrate the corresponding points between neighboring frames. (C) We can then construct a graph to represent segment evolution. A node and link denote each segment and the connection between temporally consecutive segments. (D) Flow chart of the EET algorithm. (E) All colored nodes show the ancestry of the colored node at ‘*T*→*T*+1.’ The ancestry nodes in the different frames are identified by referring to the graph shown in (C); therefore, the time course of area differences stemming from a specific segment can be identified by applying simple algebra to the ancestry node map at each time point (see Materials and Methods). The plot shows the time course of area differences corresponding to the colored ancestry. Each node includes a time course of area difference that we have defined.

The EET algorithm describes the time course of local cell morphological changes based on area differences of sequential images. We focused on the local area change, rather than the local structural change as a morphological property; therefore, EET analysis did not make clear distinctions between filopodia and lamellipodia. Subdivided regions along the cell edge boundaries are connected to the corresponding subdivided regions in the next frame, and movements of the subdivided regions are then defined by these connected subregions. Thus, the subdivided regions called “segments” are basic units in EET for quantification of morphological changes. EET describes the time course of local protrusion and retraction as follows:

1. Sequential cell edge boundaries and area differences are obtained by applying an appropriate binary filter to time-lapse microscopy images as a preprocess (see Preprocessing section). Each area difference is typically represented as a positive or negative number of pixels.
2. The traced cell boundary is divided into segments according to the area differences between two consecutive frames (see Figure 3A).
3. The boundary points of each segment are identified as “anchor points” (lowercase letters in Figure 3A).
4. The identified segments and the anchor points are projected into time and position along the cell perimeter coordinates (Figure 3B) and the segments are then colored according to the edge transitions. Simultaneously, we obtain a labeled area difference vector ***d*** according to the segments.
5. The corresponding anchor points, described in Figure 3B as pairs of open and closed circles with the same letters, are connected. Thus, the corresponding segments between neighboring time frames are identified. These connections yield a graph structure that resembles an evolutionary lineage. Figure 3C illustrates the graph structure corresponding to Figure 3A and 3B.

These connected anchor points indicate the spatial associations between neighboring time frames, and allow us to trace the corresponding regions along the time course by means of the graph structure, which represents the lineage of the segments along the time course. A flow chart of the EET procedure above is shown in Figure 3D.

It should be noted that EET defines how the ancestral segments of a certain segment at a certain time behave along the time course (Figure 3E). Because the definition of segments depends on area differences, if a cell becomes transiently immobile the subdivided regions fuse into a single, and hence integrated, edge. In such a case, integration can be avoided by an exceptional operation that maintains the anchor points during the period of immobility. This procedure keeps the spatial resolution (number of segments) of EET without artificial bias as far as used for immobile anchor points, because the average activity of a single segment and that of its divided segments are the same, and the area differences are always 0. Generally, however, continuous fluctuation is observed along the whole edge, and it is therefore possible to extract a sufficient number of subdivided regions to be analyzed. Actually, this exceptional operation is not used when analyzing the data in this manuscript. Although threshold parameters for the binarization in preprocessing affect the extraction of cell boundaries and area differences, the results of EET are consistent once the threshold parameters have been determined, even if cells show highly fluctuating behavior.

### Activity Profile

Local activity along a cell boundary is defined as the mean FRET ratio inside a circle, which has its center on the cell boundary and radius *r*. This is equivalent to using a smoothing filter with a kernel size of *r*. In EET, the representative activity of a segment is defined as the mean of local activities in the segment. We thus obtain a vector of activity ***a***, composed of the representative activities within each segment in time-lapse images. In polar coordinate-based and marker-tracking based analyses, on the other hand, local activity denotes the mean activity inside a circle, whose center is located at the intersection of a cell boundary and a radial axis or a marker position, and whose radius is *r*. Therefore, local activity is defined in EET in a manner that is conceptually similar to that in the polar coordinate-based and the marker-tracking-based methods; however, the EET analysis is performed segment-by-segment, which is statistically more stable than the polar coordinate-based and marker-tracking-based methods. The activity profile at time *N*, obtained from *N* images of time-lapse activity data, is denoted by ***a*** ^N^.

### Cross-Correlation Coefficients

We calculated cross-correlation coefficients between local area changes and activities based on the defined segments. Vector data {***a*** ^(*t*)^|*t* = 1, …, *N*−1} and {***d*** ^(*t*)^|*t* = 1, …, *N*} denote the activities and area differences of the segments extracted from the first to *N*−1 and the first to *N* frames of the same image sequence, respectively. ***a*** ^(*t*)^ and ***d*** ^(*t*)^ represent local activities at time *t* and local area differences between times *t* and *t*+1, respectively. According to Pearson's product-moment correlation coefficient, the correlation function *R*({***a*** ^(*t*)^},{***d*** ^(*t*)^},*N*) is defined as
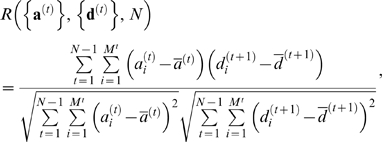
where *i* and *t* are indices for segments denoting positions along a cell boundary and time (frames), respectively, and *M^t^* denotes the number of segments at frame *t*. Note that our EET defines the activity in a segment-wise manner, and therefore ***a*** ^(*t*)^ and ***d*** ^(*t+*1)^ have the same dimensionality *M^t^*. Because the histogram of the activities in the segments was found to be approximated as a normal (Gaussian) distribution but with a heavy tail in some samples, samples whose activity exceeded 3*σ* (where *σ* is the standard deviation) were removed to avoid disproportionate influences of outliers on the correlation coefficients. When the data distributions diverged from the Gaussian, we also calculated Spearman's rank correlation coefficient, which is independent of the shape of the sample distributions, to verify the results of the Pearson's correlation coefficient. Spearman's rank correlation function *Rs*({***a*** ^(*t*)^}, {***d*** ^(*t*)^}, N) is defined as
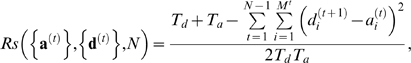
where 
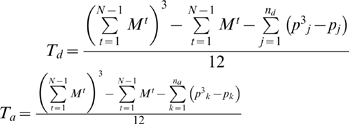
, *p_j_* is the number of rank *j* samples of {***d*** ^(*t*)^|*t* = 1, …, *N*}, *n_d_* the number of ranks in {***d*** ^(*t*)^|*t* = 1, …, *N*}, *p_a_* the number of rank *k* samples of {***a*** ^(*t*)^|*t = 1, …, N−1*}, and *n*_a_ the number of ranks in {***a*** ^(*t*)^|*t* = 1, …, *N*−1}. Because the EET calculates the cross-correlations based on segments, it is insensitive to the physical size of segments; that is, the cross-correlation coefficients indicate event-wise correlations between molecular activities and morphological changes over the whole cell edge.

### Time-Shifted Cross-Correlation Coefficients

We investigated *τ* time-shifted cross-correlation between activities and area differences to incorporate the time lag between molecular events and morphodynamics. Because the ancestry relationship between a single segment in a focused frame and segments in another frame is not one-to-one (Figure 3C), we defined the transition matrix ***A*** *^t,t+τ^* so that the *τ*-shifted area difference ***d*** ^(*t,t+τ*)^ could be defined. Because the graph structure was obtained under the basic assumption that each local event is defined in terms of ‘segment’, a morphological property, we calculate the *τ-*shifted values only for the area differences. A series of the corresponding area differences by sequential *τ*, for example, ***d*** ^(*t,t+*1)^, ***d*** ^(*t,t+*2)^, ***d*** ^(*t,t+*3)^,…, denotes the time course of edge evolution; ***d*** ^(*t,t+τ*)^ is defined below. The transition between *M^t^* segments at time *t* and *M^t+^* ^1^ segments at time *t*+1 is represented by an *M^t^×M^t+^* ^1^ matrix ***A*** *^t,t+^* ^1^, which consists of 0 and 1 denoting unconnected and connected segments, respectively, in the ancestry graph (Figure 3C). Because the column dimensionality of the transition matrix at time *t* and the row dimensionality of the transition matrix at time *t*+1 are the same as the number of segments between time *t* and time *t*−1, the transition matrix between time *t* and *t+u* can be calculated algebraically as
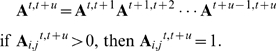
This means that each component is substituted by one if the matrix calculation results in a positive value. Corresponding area changes from time *t* to time *t+τ* are then expressed as:
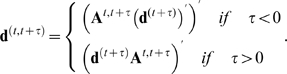
The *i*-th element of ***d*** ^(*t,t+τ*)^ denotes the summation of area differences among the segments at *t*+*τ*, which are ancestral to the *i*-th segment at *t,* according to the ancestry graph. In Figure 3C, for example, ***d*** ^(*T*)^ = {la, ab, br} and ***d*** ^(*T+*1)^ = {lc, cd, de, ef, fr}, where each element in the sets denotes an area difference (typically, a number of pixels). The transition matrix is given by:
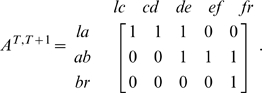
Then, ***d*** ^(*T+*1,*T*)^ = (**A** ^T,T+1^ (***d*** ^(*T+*1)^)’)’ = {lc+cd+de, de+ef+fr, fr}, where the addition is applied to the area difference values. Based on these time-shifted corresponding area differences, a one-to-one relationship between the segments in different frames is constructed. The cross-correlation coefficient with a time-shift of τ is thus obtained by calculating *R* ({***a*** ^(*t*)^}, {***d*** ^(*t, t+τ*)^}, *N−τ*).

### Preprocessing

In this study, cell boundaries and area differences were all extracted from fluorescence time-lapse images. To emphasize the cell edges, the images were filtered with an unsharp mask (implemented by the image-processing software MetaMorph [Universal Imaging, Sunnyvale, CA]), which subtracts a low-pass filtered and scaled image from its original image. The Gradient Anisotropic Diffusion filter [29],[30] was then applied to smooth edge boundaries for complex cell shapes. After the filtering step, the intracellular and extracellular regions were segmented using the global threshold determined for the first frame. The cell boundary was extracted directly from the outline of the thresholded images. Typically, the extracted cell boundaries were distorted when edge extraction was applied to threshold regions with one-pixel width, such as thin spikes. To avoid this, each pixel in a thresholded image was divided into sub-pixels before extraction of boundaries. Boundary extraction was then executed for each binary image at a sub-pixel resolution. We did not apply spline fitting in EET or polar coordinate-based analysis to avoid spoiling steep edge structures with filopodium-like thin shapes. Area differences were also extracted from the thresholded images. Increased areas were determined by subtracting the current frame from its next frame, while decreased areas were determined by subtracting the next frame from the current one. Most of these procedures, including EET and cross-correlation analysis, were implemented by Matlab (The MathWorks, Natick, MA).

### Time-Lapse FRET Imaging

For this study, we used neurite outgrowth of rat pheochromocytoma PC12 cells as an example of cells displaying complex morphological dynamics, while random migration of human fibrosarcoma HT1080 cells was used for analysis of the cross-correlation between morphological changes and Rho GTPase activity. PC12 cells were plated on polyethyleneimine- and laminin-coated 35-mm glass-base dishes (Asahi Techno Glass, Chiba, Japan), and then transfected with pRaichu-1011x encoding Rac1 FRET probe. One day after transfection, the cells were stimulated with 50 ng/ml NGF in phenol red-free Dulbecco's modified Eagle's medium/F12 containing 0.1% bovine serum albumin for 48 h to induce neurite outgrowth. HT1080 cells were transfected with pRaichu-1011x, pRaichu-1054x encoding a Cdc42 FRET probe, or pRaichu-1294x encoding RhoA FRET probe and, after 24 h, cells were plated on collagen-coated 35-mm glass-base dishes. The medium was then changed to phenol red-free Dulbecco's modified Eagle's medium/F12 containing 10% fetal bovine serum, overlaid with mineral oil to prevent evaporation, and image acquisition was started. The cells were imaged with an inverted microscope (IX81 or IX71; Olympus, Tokyo, Japan) equipped with a cooled charge-coupled device camera (Cool SNAP-K4 or Cool SNAP-HQ; Roper Scientific, Duluth, GA), and a laser-based auto-focusing system at 37°C. The filters used for the dual-emission imaging were purchased from Omega Optical (Brattleboro, VT): an XF1071 (440AF21) excitation filter, an XF2034 (455DRLP) dichroic mirror, and two emission filters (XF3075 [480AF30] for CFP and XF3079 [535AF26] for FRET). The cells were illuminated with a 75-W xenon lamp through a 6%, 10% or 12% ND filter and viewed through a 60× oil-immersion objective lens (PlanApo 60×/1.4). The exposure times for 2×2 or 3×3 binning were 400 or 500 ms for CFP and FRET images. After background subtraction, FRET/CFP ratio images were created with MetaMorph software, and the images were used to represent FRET efficiency. Further details of microscopy and sample preparation can be found in previous reports [26],[27].

### Permutation Test

We executed a permutation test between positive (6 min), negative (−6 min) and non time-shifted correlations according to the following procedure. Letters/numbers in bold fonts represent vectors.

1. Correlations are obtained at positive (**CP**), negative (**CN**) and non- (**C0**) time-shifts by analyzing cells with EET, polar coordinate-based and marker-based analyses. There are three kinds of labels: positive, negative and zero, for each correlation vector.
2. For each pair of the three labels:
3.
  - Calculate the difference between the two correlation vectors, for example, the difference between positive and negative labels is given as **D_P_N** = **CP**−**CN**.
  - Resample permutated differences of correlations (for example **D_P_N^per^**) by randomly (but systematically and exhaustively in this case, see the example below) changing the plus/minus sign of each element in the original difference vector.
  - Calculate the rates (i.e., permutation *p*-value) of permuted difference vectors with a lager mean value than that of the original difference vector among all the permutated difference vectors.
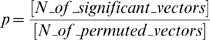

  -
*N_of_significant_vectors* denotes the number of permuted difference vectors, whose mean is larger than the mean of the original difference vector.
*N_of_permutated_vectors* denotes the number of all the permuted difference vectors obtained in step 2(A).
4. Calculate the permutation *p*-values above for all pairs of **CP**, **CN** and **C0**.

For example, if we have **CP** = [0.6 0.4 0.6] and **CN** = [0.3 0.5 0.4], then **D_P_N** = [0.3 −0.1 0.2]. Each permutated difference vector is an element of the set of possible ones: **D_P_N^per^**∈{[0.3 0.1 0.2], [0.3 0.1 0.2], [0.3 0.1 −0.2], [−0.3 0.1 0.2], [−0.3 −0.1 0.2], [−0.3 0.1 −0.2], [0.3 −0.1 −0.2], [−0.3 −0.1 −0.2]}. Owing to the independence assumption of a sign-change between elements, the permutation (null) distribution is simply obtained by arranging all the possible sequences, whose number is 2^3^ = 8 in the above example, with uniform probability. The permuted difference vectors whose mean is larger than that of the original difference vector are thus {[0.3 0.1 0.2], [0.3 −0.1 0.2]} and number 2. In this particular example, the permutation *p*-value is then given as *p* = 2/8 = 0.25. If this *p*-value is smaller than a specified significance level (usually 5%), the difference between **CP** and **CN** is said to be significant. In the case of two-sided permutation test, the significance level is simply divided by 2.

## Results

### EET Profiling for Branching Neurites of PC12 Cells

**Figure 4:**
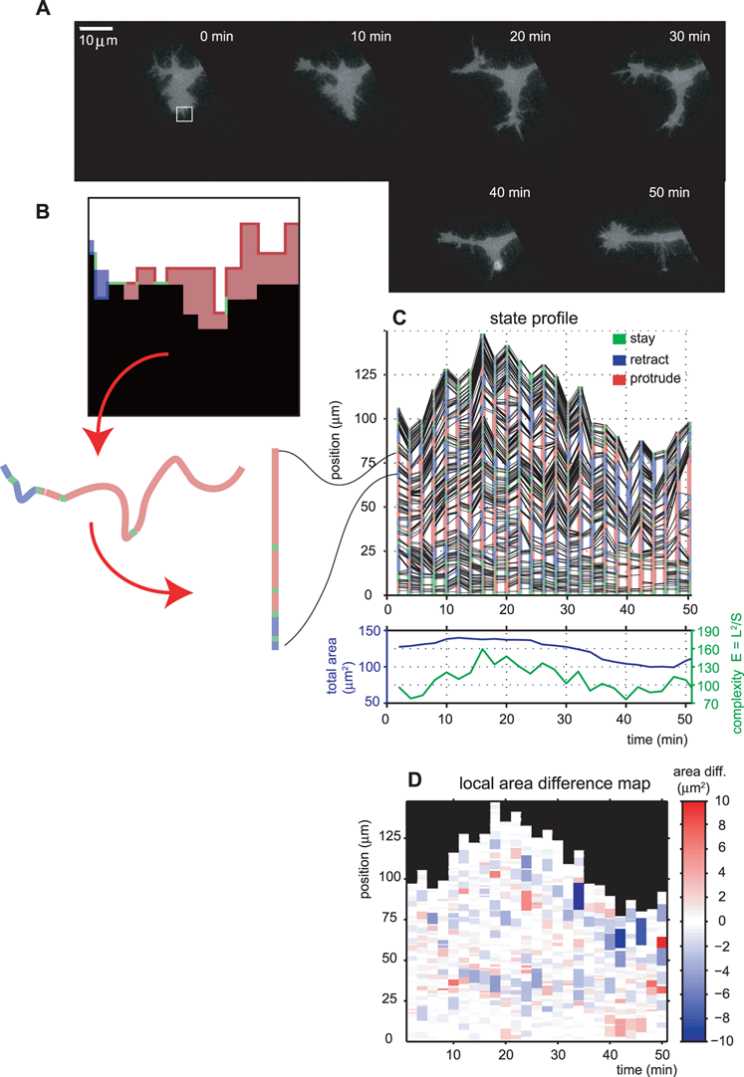
The EET profile of a branching PC12 cell. (A) Time-lapse fluorescence images of a PC12 cell. (B) Expanding, retracting, and stationary regions of the cell edge boundary in the subsection of (A) (white square) are colored red, blue and green, respectively. Each colored region along the cell edge corresponds to a single segment in panel (C). Red arrows show the correspondence between colored regions in (B) and segments in (C). (C) The cell boundary state profile of (A), in which each segment is colored red, blue and green according to the status of expansion, retraction and stasis, respectively. Black lines connect the corresponding anchor points to represent the correspondence between subdivided regions in successive frames. The plot shows the total cell area and complexity {(total cell boundary length)^2^ /(total cell area)} of the cell. Note that the total cell area and the total length of the cell boundary are highly independent. (D) Local area difference map of (C), in which the magnitude of area difference for each segment is depicted by a color gradation from protrusion (red) to retraction (blue).

We applied EET to branching PC12 cells to validate its usefulness for quantifying complex cell morphological changes. As shown in Figure 4A, the PC12 cells extended their neurites with branches after treatment with NGF. A time-lapse series (1-min intervals) of the images was trimmed to help maintain visual correspondence with EET profiles because large image sizes may make the visual inspection difficult. We chose the branching region to verify the utility of EET for the complex cell shape. Next, following the EET procedure, we determined the profiles of edge boundary states, as depicted in Figure 4C, in which red, blue and green colors denote protrusive, retractile and pausing states of the cell edge boundary, respectively. Black lines connect the anchor points (see Materials and Methods), and represent the corresponding segments and subdivided regions. Small fragments of the segments show spatially independent and transient behaviors of the edge evolution and contraction, while long segments represent simultaneous occurrence of edge evolution and contraction in neighboring regions during the time lapse. We also monitored global changes in cell morphology using total area and complexity (bottom of Figure 4C), together with the state profiles, because the state profile by itself does not illustrate the global characteristics of cellular morphodynamics. The monitored total areas and complexity represent the balance between the length of the cell edge boundary and the total area. These values will help to identify rough images of morphological changes. To visualize the dynamics of local area differences by EET, an area difference map was constructed as shown in Figure 4D. Despite the complex morphological changes, EET was successful in quantifying detailed local area changes and preserving the positional correspondence among the subdivided edges. For example, the white squared area in Figure 4A showed a slight extension until 20 min and then retraction between 30–50 min; this corresponds to the region in the state profile starting from 60–80 mm (ordinate) at 0 min (abscissa) (Figure 4C). This quantification and visualization method reduces the difficulty in dealing with time-lapse image data by summarizing the morphodynamic characteristics into two-dimensional state profiles.

### EET Profiling for Rho GTPase FRET Images of Motile HT1080 Cells

**Figure 5:**
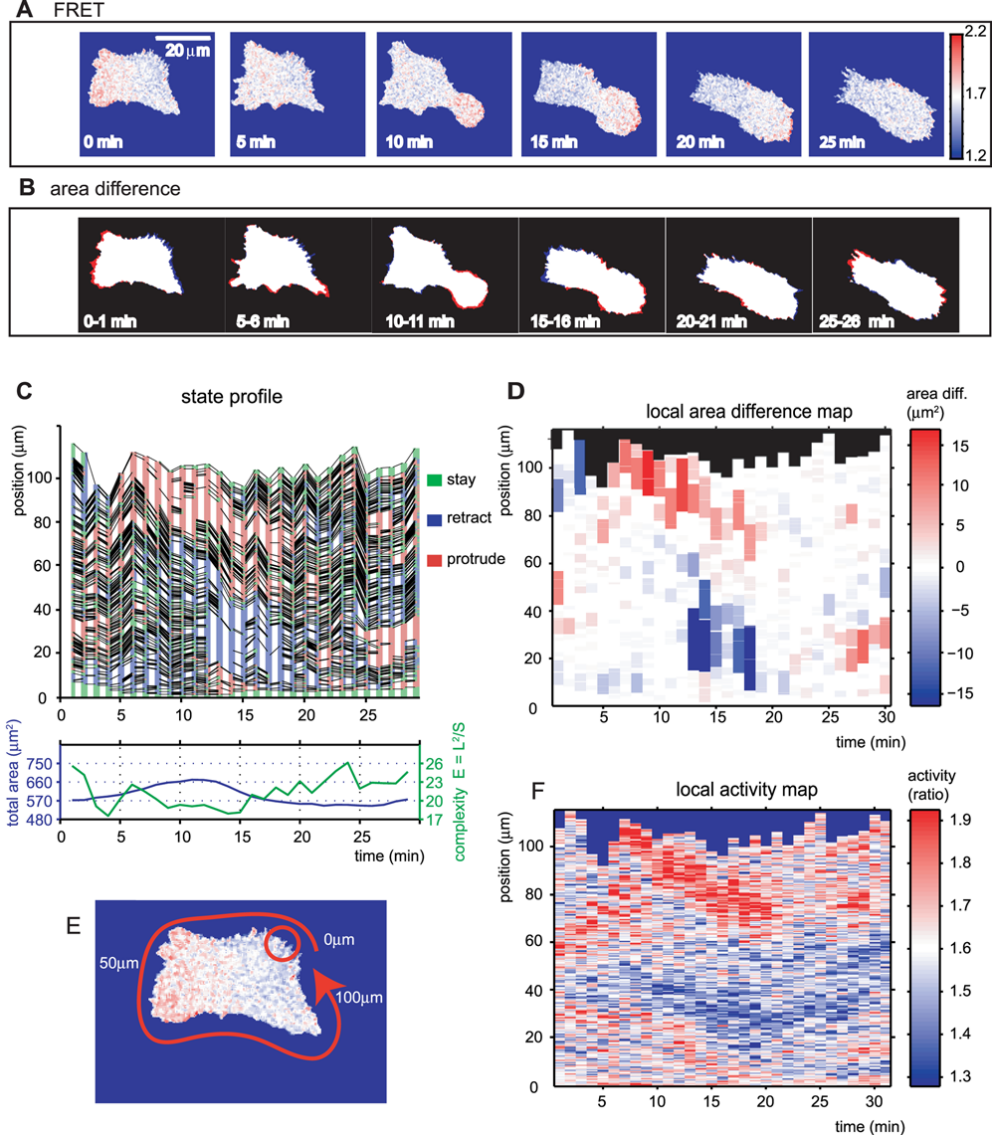
FRET and area difference images, and EET profiles, of a motile HT1080 cell. (A) Time-lapse FRET images of an HT1080 cell. The colored bar illustrates the FRET/CFP ratio, which is assumed to indicate Rac1 activity. (B) Area difference images are acquired by subtracting neighboring frames (see Materials and Methods). Red and blue denote expansion and retraction, respectively. This cell moves by approximately 60 µm in 60 min and many of other active cells are free to move to a similar extent. (C) Edge state profile for the same motile HT1080 cell, and (below) global characteristics (total area and cell complexity). (D) Area difference map for (C). (E, F) We define the local activity as the mean of the intensities inside a circle of radius *r*. (E) A schematic view is shown with the circle (red circle) and direction of the position axis (red arrow) in (F). Although the length of *r* was chosen arbitrarily, this does not substantially affect the result (see Figure S1). The extracellular region is excluded for the mean calculation. In (F), the local activities along the cellular edge are mapped into a time-position representation as in (D) (see Materials and Methods). The colored bar shows the FRET/CFP ratio. Spatio-temporal activity patterns resemble those in the local area difference map.

Because previous studies have shown the localization of GTPase activities at peripheral regions [26], we applied EET to motile HT1080 cells to further quantify the relationship between local morphological changes and local GTPase activity. First, we imaged motile HT1080 cells with a 1-min time-lapse. Figure 5A shows a series of FRET/CFP ratio images of a single motile HT1080 cell expressing Raichu-1011x (Rac1 probe), and the FRET efficiency is shown in pseudo colors. Based on a previous study indicating a correlation between FRET efficiency and Rac1 activity [26], we assumed that the Rac1 activity should be well represented by the FRET efficiency. The time-lapse images reveal the wandering behavior of the HT1080 cell and a spatio-temporal activity pattern of Rac1 within the cell. To emphasize the protruded and retracted areas in consecutive frames, each image was first transformed into a binary image by extraction of the cell and background regions. The consecutive subtracted images were then obtained frame by frame, and the protrusion and retraction areas were colored in red and blue, respectively (Figure 5B). As reported previously, the coincidence of morphological changes with increases in Rac1 activity was seen by comparing the FRET and subtracted binary images (Figure 5A and 5B).

Next, we applied EET to precisely examine the spatio-temporal relationships between morphological changes and GTPase activities in motile HT1080 cells. As with PC12 cells (Figure 4C and 4D), the state profile and local area difference map were acquired (Figure 5C and 5D). Simultaneously, we acquired the local activity map (Figure 5F) based on segment-wise local activity (Figure 5E, Materials and Methods). This time-position map of the local GTPase activity corresponds to both the state profile and the local area difference map (Figure 5C, 5D, and 5F). There appeared to be similar patterns between the local area difference map and the local activity map. The area difference map revealed chunks of persistently protruding or retracting regions at the cell periphery, while the activity profile revealed spatially and temporally associated activity patches at the cell boundary, suggesting that their dynamics correlated with each other.

Visual inspection of the local area difference map (Figure 5D) and local activity map (Figure 5F) helped us to detect patterns of cell morphology and GTPase activity. The upper left area of Figure 5D shows that formation of large lamellipodia (between 6–20 min) was preceded by the local retraction of the cell edge, and this retraction-extension pattern was also identified in other cell types (data not shown). Cell edge retraction has the potential to induce tension-dependent development of molecular activities involving Rho GTPase signaling [31]. Our data are consistent with this mechanosensory function and provides a possible mechanism for interactions between morphological changes and molecular signaling. On the other hand, the large retraction between 12 and 18 min (Figure 5D) was preceded by a local decrease in Rac1 activity (blue zone in Figure 5F at 10–12 min) and similar patterns were also observed in other cells (data not shown). Potentially, the local decline in Rac1 activity may contribute to the subsequent cell-edge retraction. In addition, in contrast with the morphological changes, the local activity map revealed that the GTPase activity changed moderately at the same position. This moderate change may help maintain the stability of the polarity.

### Distributions and Time-Shifted Profiles of Morphological Changes and GTPase Activities

**Figure 6:**
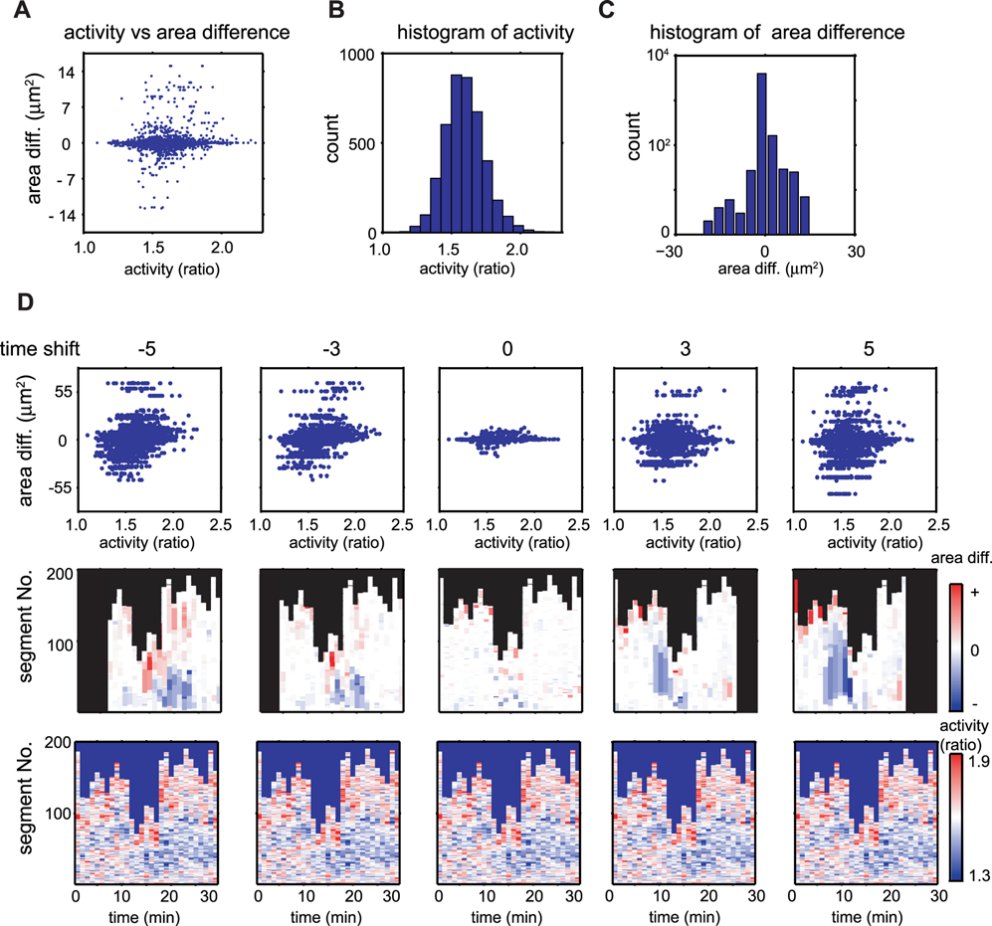
Local activity and local morphological change distribution properties. (A) A scatter plot of the local activity and area difference of each segment. Each point represents the local activity and area difference of a single segment identified by EET. The overall property of all the segments in the dataset is portrayed, excluding temporal and positional information. (B) Histogram of GTPase activities (YFP/CFP ratio) approximated by Gaussian distribution. Vertical and horizontal axes denote the number of segments and local activity within each segment, respectively. (C) Histogram of area differences in each segment. Zero values occur frequently because the majority of edge segments do not move. (D) Time-shifted relationship between local area differences and GTPase activity. The top panels show the time-shifted scatter plots of the local area difference and the GTPase activity. Each point represents the mean local activity and summation of the area difference of the ancestry segments (see Materials and Methods). The same data are exhibited in different scales in (A) and (D) depending on the context; that is, (A) shows the detailed distribution of the activities and the area differences to provide clear comparisons with (B) and (C), while the upper panels in (D) show the differences between various time-shifts. The middle panels show the time-shifted area difference maps of the corresponding scatter plot in the top panel. The colored areas denote summation of the corresponding area differences at each shifted time. The numbers of columns are reduced with time-shifts because time-shift produces non-corresponding frames. GTPase activity maps without time-shifts are displayed in the bottom panels to illustrate their relation with the corresponding time-shifted area difference maps. Note that all activity maps in the bottom row are identical. A linear correlation appears with negative time-shifts (time-shift: −5 and −3 in the top scatter plots), whereas no correlation is observed with positive time-shifts (time-shift: 3 and 5 in the top scatter plots).

We further investigated this spatio-temporal cross-correlation between morphological changes and Rho-family GTPase activity. First, we summarized their statistical characteristics to examine the cross-correlation. Figure 6A shows a scatter plot of the local activity and the local area difference for all identified segments. Because there were no non-linear relationships in this plot, we considered that common statistical analyses could be applied to these data. Next, we examined the histograms of the activity and area difference and found that the activities had a Gaussian distribution (Figure 6B); heavy tails were observed in some samples, but not in the area differences (Figure 6C). Although the activity histograms of a few samples exhibited one or two minor peaks in addition to the major peaks (data not shown), we assumed that they could still be approximated by Gaussian distribution for simplicity; in subsequent analyses, we used both Pearson's product-moment correlation coefficient and Spearman's rank correlation to confirm the cross-correlation data.

We next examined the effects of time-shifts on cross-correlation between the activity and the area difference. The graphical structures of EET profiles display local area differences in the corresponding time-shifted segments. The middle panels of Figure 6D show time-shifted local area difference maps with various time-shift values. Different patterns appeared on the area difference map depending on the time-shifts, showing that the correlation changes depend on the time-shift values. The scatter plots of activity without time-shift against time-shifted area differences show a linear relationship for negative values of the time-shift (Figure 6D upper).

### Cross-Correlation between Local Morphological Dynamics and Local GTPase Activities

**Figure 7:**
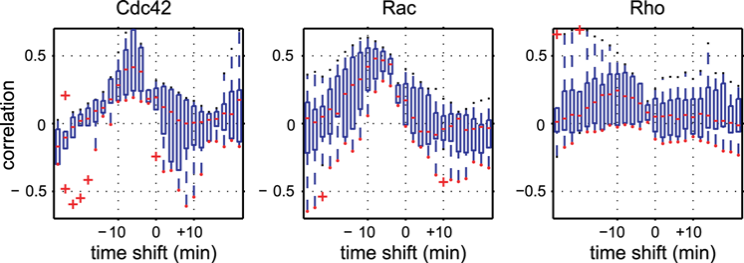
Time-shifted cross-correlation between GTPase activities and area differences. Rho family small GTPases Cdc42, Rac1 and RhoA were analyzed in terms of the time-shifted cross-correlation. We examined several cells for each GTPase. Each boxplot shows the first quartile (bottom of the box), third quartile (top of the box), median (red line) and outliers (red plus marks) for several cells (N = 9 for Cdc42, N = 6 for Rac1 and N = 6 for RhoA). Where there were no outliers, a red dot is shown at the bottom of the whisker. For Cdc42 and Rac1, the time-shifted correlation is significantly increased with negative time-shifts (results of the permutation test are shown in Table S1).

We calculated time-shifted cross-correlations between the local activities of Cdc42/Rac1/RhoA and local morphological changes, as shown in Figure 7. As expected, there were strong correlations between Cdc42/Rac1 activities and morphological changes, but the peaks of the correlation coefficients were slightly time-shifted. Moreover, and surprisingly, the peaks indicated that the local morphological changes preceded changes in local activity, which can be seen in Figure 6D. We confirmed statistical significance of the difference between negative (−6 min), zero and positive (+6 min) time-shifts by performing permutation tests (see Table S1). The number of samples used to calculate the cross-correlations was sufficiently large (see Figure S2 and Figure S3). Although there are some conspicuous morphological events seen in the EET profile (Figure 5C), such as the protrusion around 6–16 min and the retraction around 12–18 min, the cross-correlation based on the EET analysis was designed to be robust against such local events arising in limited sites in the cell. In this specific case of Rac1 activity in HT1080 cell, our finding that the cross-correlation profile is highly correlated with minus time-shift values is unchangeable, even when these conspicuous morphological events are replaced by normal morphological events (see Figure S4). Note that the Spearman's rank correlation also reduces the bias effect of large values (events) on statistical values.

The results do not appear to be intuitive with regard to the causal relationship between morphological changes and molecular signaling; upstream molecular signaling should control downstream morphological changes, for example via actin reorganization, adhesion and/or retrograde flow. In the cases of both Rac1 and Cdc42, the time-shifted correlations showed that morphological change preceded local GTPase activity. Cdc42 activity, in particular, showed large deviations when the preceding time-shifts were short, and the correlation decayed steeply when the time-shifts were longer. Rac1 activity, on the other hand, elicited small deviations and the decay of the correlation was less steep when the preceding time-shifts were longer. It should be noted that the time-shifted correlation generally approaches zero over long time-shifts owing to an increase in the number of connections between the original segment and time-shifted segments (see Figure 3E). This reflects a weakened relationship, i.e., not one-to-one but one-to-multi relationship between the original region (segment) and its time-shifted regions (connected segments). However, this weakened relationship does not imply a decrease in the reliability of calculations of time-shifted coefficients by making vague relationships between time-shifted segments, but instead represents the natural dilution of the correspondence between an original region and its time-shifted regions.

We further examined the spatial property of the relationship between GTPase activity and morphology change by comparing the original EET profile with rotated (see Figure S5A) and permutated (see Figure S5B) segments of EET profiles. EET profiles of rotated segments showed a decreased correlation with increased rotation (see Figure S5C). Because the segments have a range of lengths along the cell edge, EET did not directly show an exact proximity. However, it showed the significance of the locality of morphodynamic regulation signal. The signal locality dependency was also shown by a lack of correlation of the permutated segments profile with EET.

### Comparison of EET with Polar Coordinate-Based Analysis

**Figure 8:**
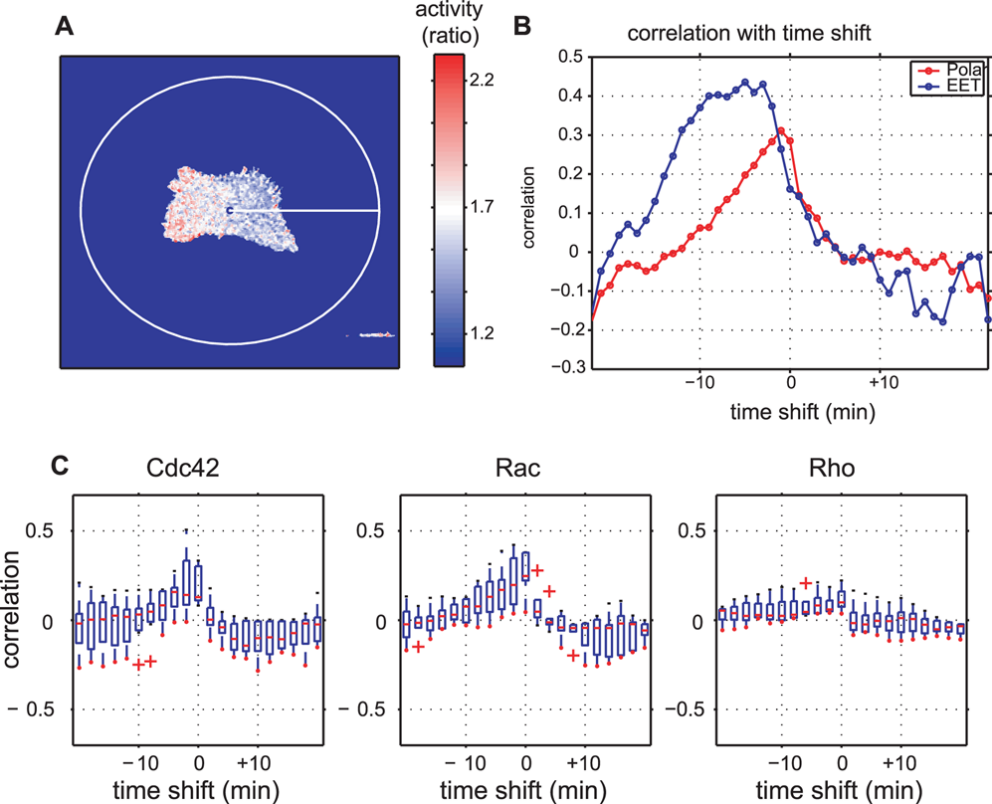
Comparison of morphodynamic analysis by EET with polar coordinate-based analysis. (A) Polar coordinate-based analysis was performed by setting the origin of coordinates at the mean mass center of the binary images. (B) Time-shifted cross-correlation analysis by polar coordinates and EET for the cell depicted in Figure 5. Both of the correlation profiles show positive correlations with negative time-shifts and low correlations with positive time-shifts. However, EET yields a higher correlation than the polar coordinate-based method for the negative time-shifts. (C) The same cells in Figure 7 were also analyzed by polar coordinate-based analysis. All panels show similar shapes to that in Figure 7; however, peaks in Cdc42 and Rac were lower with polar coordinate-based analysis than with EET.

We compared EET analysis to polar coordinate-based analysis to further prove the utility of EET. We first performed polar coordinate-based analysis to the cell in Figure 5 for direct comparison with EET (Figure 8A). The polar coordinate-based analysis produced time-position maps of local activities and local morphological changes that were similar to the activity map and area difference map of EET (see Figure S6). As for EET, local activity was determined as a mean value within an ROI, which was a circle of radius *r*. We used the same *r* value for EET and the polar coordinate-based method. Both analyses produced similar maps (see Figure S6), and time-shifted cross-correlations were then calculated (Figure 8B). Both of the time-shifted cross-correlations showed similar patterns for the timing between local morphological changes and GTPase activities (Rac1), i.e., a high correlation with negative time-shifts and a low correlation with positive time-shifts. However, the EET analysis showed a higher correlation than that with the polar coordinate-based analysis at the time-shifts of −3 to −20 min.

A similar tendency was observed when a population of the cells in Figure 7 was analyzed by the polar coordinate-based method (Figure 8C). The averaged peaks of cross-correlations obtained by the polar coordinate-based analysis were substantially lower than those obtained with EET, particularly for Cdc42 and Rac1 (Figures 7 and 8C). Permutation tests revealed significant differences between the time-shifted cross-correlations by the polar coordinate-based analysis (see Table S1). This might be due to the relatively large correlation values at the time-shift of zero. However, the variances were small, and the correlations prominently decreased when the time-shift value was far from zero. Statistical tests generally showed significant differences between two groups when the variance of each group was small. Here, the small variances in the correlations are likely to be obtained by averaging a large number of samples with small values, and the small values may be due to inconsistency in position alignment between different frames. Note that the polar coordinate-based analysis acquired a large number of samples at 1-degree intervals (i.e., 360 samples in each image) from a single cellular edge and that adjacent samples were likely to have similar values because of physical edge continuity. Our EET implemented the sensitivity to detect correlations between activities and morphological changes by maintaining a consistent position between consecutive frames in terms of segments. Thus, we believe that the correlation peak at the time-shift of zero, obtained by the polar coordinate-based analysis, could be an artifact stemming from position misalignment.

### Comparison of EET with Marker-Tracking-Based Analysis

**Figure 9:**
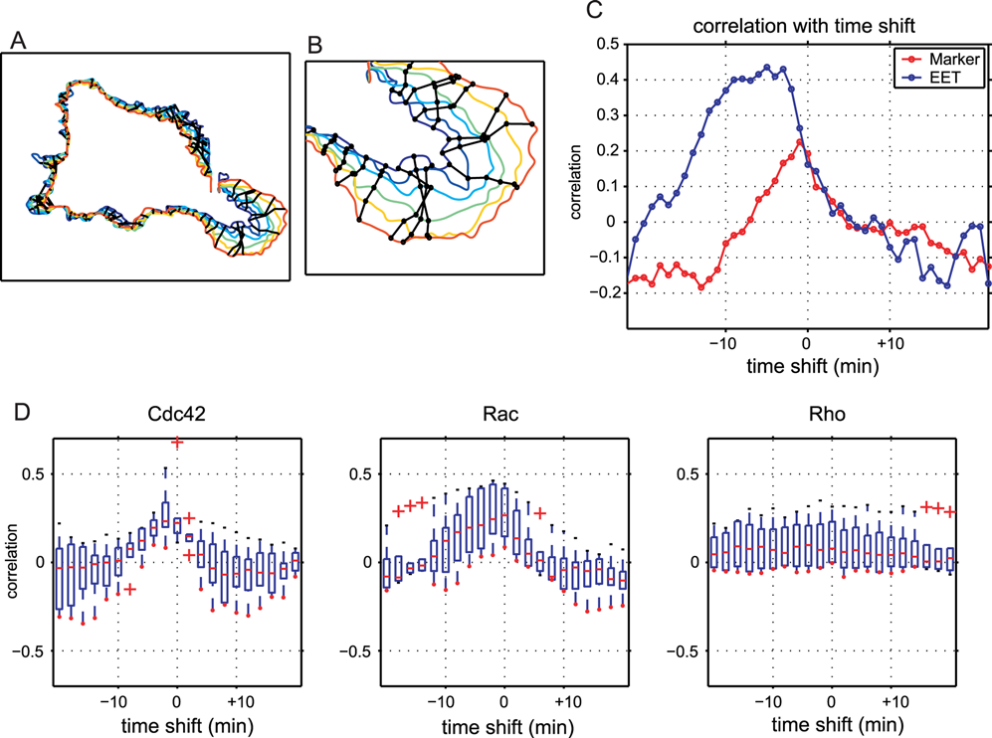
Comparison of morphodynamic analysis by EET with marker-tracking-based analysis. Marker-tracking-based analysis was performed using virtually-defined markers, and their movements perpendicular to the cellular edge were measured. (A) Cellular edges changing with time (blue: 6 min; indigo: 7 min; light blue: 8 min; green: 9 min; yellow: 10 min; red: 11 min). The cell analyzed was the same as that used in Figure 5. Black lines show traces of virtually defined markers. (B) Closed subsection of the lower right area in (A). Black dots show the positions of the markers. The uniform distribution of the markers (dots on the blue line) changed into a non-uniform distribution accompanied by persistent protrusion (dots on the red line). (C) Time-shifted cross-correlation analysis by the marker-tracking-based method and EET on the cell in Figure 5. Both of the correlation profiles show strong positive correlations in negative time-shifts and weak correlations in positive time-shifts. However, EET yielded higher correlations than the marker-tracking-based method in the negative time-shifts. (D) The same cells as in Figures 7 and 8 were also analyzed by the marker-tracking-based analysis. All three panels show similar shapes to those in Figures 7 and 8, but the peaks in Cdc42 and Rac were lower with marker-tracking-based analysis than with EET.

We also compared EET analysis with simple implementation of marker-tracking-based analysis. In this marker-tracking-based analysis, virtually defined markers were aligned uniformly along the spline-fitted cellular edge in the first frame of time-lapse FRET images. Then, the movements of markers in the direction perpendicular to the cellular edge during a single time-frame were measured according to the current marker position and the intersection of the perpendicular axes of the current cellular edge and the next cellular edge (Figure 9A and 9B). Figure 9A and 9B show time-lapse cellular edges of the same cell as in Figure 5, colored from blue (6 min) to red (11 min), with virtually defined markers (black dots) and movements of the markers (black lines). Topological violations of the markers (crossing the black lines) are indicated in Figure 9B, which is probably due to the highly complex morphological changes in the edges. Such complex changes could affect the marker movement maps (although the map obtained by the marker-tracking-based method was comparable to that obtained by EET and by polar coordinate-based analysis; see Figure S6), but our statistical analysis was not affected. Instead, the changes in marker distribution from a uniform (black dots on the blue line in Figure 9B) to a non-uniform alignment (black dots on the red line in Figure 9B) would have non-negligible influences on the time-shifted statistical analysis (e.g., Figure 1C). As with EET and the polar coordinate-based method, the local activity was determined as a mean value within an ROI, which was a circle of radius *r*. We used the same *r* value in EET, the polar coordinate-based and the marker-tracking-based methods. All analyses produced similar maps (see Figure S6), and time-shifted cross-correlations were then calculated (Figure 9C).

The time-shifted cross-correlations in Figure 9C show lower correlations at the negative time-shifts compared with EET. The marker-tracking-based analysis produced similar patterns of time-shifted cross-correlations for Cdc42 and Rac1 (Figure 9D) and the permutation tests revealed significant differences between the correlations at zero, and the negative and positive time-shifts (see Table S1). Similar to the polar coordinate-based method, the marker-tracking-based analysis revealed weaker characteristics in the time-shifted cross-correlations. This seems to result from biased sampling by the non-uniform marker distribution caused by morphological changes, which can be seen in Figure 9B. Thus, we suggest that the marker-tracking-based analysis has undesired affects on the statistical analysis, particularly when the cellular edge has a persistent deforming property.

## Discussion

We have developed an algorithm called EET, which describes changes in cell morphology using time-lapse live cell imaging. Spatio-temporal area difference maps revealed morphodynamic properties as patterns of extension and retraction, and the correspondence between time-shifted segments, achieved using anchor points, ensured that the related subdivided edges were connected between time-shifted frames. Therefore, EET effectively accounts for complex morphodynamics that include persistent extension or retraction, and arborization. This property is realized by the graphical representation of edge evolution, and ensures EET is suitable for depicting changes in cell shape, such as the branching that occurs during neural development. Application of EET to the extending neurites of PC12 cells provided a clear evidence of its utility by precisely revealing the persistent protrusion and retraction patterns. Besides, a second application to motile HT1080 cells illuminated distributions of local area differences and corresponding local activity of GTPases. Although the graph structure itself potentially generates biases when correlating the one-to-multi segments between temporally distant frames, we confirmed that our results were consistent even when we obtained our result differently by associating the area change in each segment with the average molecular activities over the corresponding segments (see Figure S7). Because cellular morphological changes have probabilistic characteristics [32], the statistical analysis approach used here is a powerful tool for exploring the nature of dynamic processes in cellular behaviors.

It has been established that Rho-family GTPases (Rac1, Cdc42 and RhoA) play key roles in morphological changes through cytoskeletal reorganization [19], [33]–[35]. Furthermore, previous FRET imaging studies have shown that these GTPases are exquisitely regulated spatio-temporally [25],[26],[28],[36]. In this study, we obtained additional results with EET analysis. In particular, the activities of Rac1 and Cdc42 were localized around the peripheral regions and strongly correlated with the preceding changes in the local area, while the local activity of RhoA was only weakly correlated with changes in the local area. The activity of Cdc42 immediately preceding to the activity of Rac1 is consistent with earlier finding, suggesting that Rac1 is activated by active Cdc42 [37], while the difference in time-shifted cross-correlations between RhoA and Cdc42/Rac1 (Figure 7) would supports the existence of feedback loops common to Rac1 and Cdc42. However, the relationship between RhoA activity and morphology remains controversial [25],[38]. Quantitative analyses in different experimental conditions will clarify this issue. Our results, however, should prompt further investigation of the role of GTPase in regulation of morphodynamics, because this challenges the hypothesis that Rac1 and Cdc42 promote extension of lamellipodia or filopodia, respectively.

The precise mechanism by which local area changes precede local activity around the cell boundary remains unclear from our current analysis. However, we speculate four possible mechanisms based on our results. The first explanation is the existence of upstream signaling molecules that regulate extension in parallel with GTPase activity. If the reactions of the signaling cascades involved with extension are faster than those linked to GTPase activation, extension could precede GTPase activity. In this respect, it would be interesting to conduct a study similar to the current one for PI3K, which activates many signaling molecules including Rac1 activators [39].

The second explanation is that protrusion site-specific stimulation activates the GTPases. There are several mechanisms by which physical force can be converted into biochemical responses [40], and a theoretical study has suggested that signaling activity might be affected by cell shape [41]. In addition, we have shown that there is a positive feedback loop from actin polymerization to Rac1/Cdc42 activation via PI3K [39]. Therefore, it is possible that the detected increase in Rac1/Cdc42 activation was, in fact, secondary to actin polymerization at the protruding regions.

The third possibility is that signaling crosstalk regulates the timing of extension and retraction [42]. If the GTPase activity induces extension and also activates factors that promote edge retraction, the peak GTPase activity appears to be delayed with morphological changes by balancing with activated retraction promoter.

The fourth possibility is the existence of different mechanisms for cell edge extension. EGF-stimulated initial protrusion in MTLn3 rat adenocarcinoma cells is caused by cofilin activation and severing of F-actin, which is coincident with actin polymerization and formation of lamellipodia [43]. On the other hand, Rac1-dependent edge expansion is followed by stabilization of the protrusions [44]. Further investigations will enable us to determine which hypothesis (including coexistence) is most likely with the observed phenomena. In addition, the effects of the dynamics in the perpendicular axis such as changes in cell thickness and volume should be determines, because our results are restricted to the horizontal dynamics. Probe-related mechanisms should also be considered carefully; for example, the difference in the expression levels between the FRET probes and the endogenous Rho GTPases might affect the timing and dynamics of activation of GTPases.

Quantitative analysis of live cell microscopy images is invaluable for better understanding of the dynamic properties of processes such as chemotaxis and development. Such quantitative data can go beyond descriptions of the dynamic features of cellular behavior to serve as a scaffold for theoretical study and to enhance system-level understanding. Based on quantitative data acquired by polar coordinate-based analysis of neurons, for example, Betz et al. discussed a bistable stochastic process derived from velocity histograms and calculated potential distribution [32]. Therefore, connecting modeling studies with quantitative experimental studies has the potential to yield breakthroughs in system-level understanding of cellular functions [45]–[47]. The EET method allows us to quantify details of morphological dynamics of cells. Moreover, it also enables to investigate the spatio-temporal relationship between morphological dynamics and local molecular signaling dynamics. Further application of EET to other signals, e.g., different species of GTPases such as Ras and upstream signals of Rho GTPases such as PI3K, and also to localization of actin should shed light on some of the dynamic and complex properties of regulation of the morphological/migratory systems in cells.

## Supporting Information

Figure S1 Effect of radius. (A) Activity maps with several radius lengths are shown in the upper panels. Maps become increasingly blurred with increasing radius due to the averaging effect within each circle. Histograms of activity for each segment (lower panels) become sharper with increasing radius because an increase in the circle size leads to both a decrease in the population of outliers and an increase in the population of mean segments. A few zero-activity segments occur as a result of failure of cell edge tracing in preprocessing. (B) Time-shifted correlations for several radii. Qualitatively similar profiles are shown for four radii. To precisely determine the length of the radius, however, there is a trade-off between noise reduction and retention of map clarity. For subsequent analysis, we set the length at *r* = 7.5 µm.
(1.80 MB EPS)

Figure S2 Sample numbers with time-shifts. Cross-correlation between local activity and area difference (black line) and sample number (red line) was plotted against time-shift. Because we executed statistical analysis segment-wise, the sample number for calculating the cross-correlation is the same as the number of all segments ∑^N−1^_t = 1_ *M* ^t^. Although the sample number decreases as the time-shift increases, a statistically sufficient number of samples (more than 2000) were obtained with EET.
(0.93 MB EPS)

Figure S3 Effect of time-lapse length. (A) We examined how the time-shifted correlation behaves when the time-lapse length (i.e., number of images) changes. The time-shifted correlation was calculated for different time-lapse values with the same cell. We made a series of different time-lapse images by extracting selected images. For example, from seven frames of 1-min time-lapse images {1 2 3 4 5 6 7}, we extracted 2-min {1 3 5 7} and 3-min time-lapse images {1 4 7}. All profiles show quantitatively the same behavior; that is, a high correlation for around −7 to −13 min time-shifts and no correlation for positive time-shifts. (B) The relationship between various time-lapse values (1 to 3 min) and sample numbers (segment numbers) decreases with increasing time lapse. Longer time lapses tend to show higher correlation owing to the tight relationship between persistently extending cell peripheries and GTPase activities. On the other hand, we should choose a time-lapse length considering the decrease in the sample number, which may reduce the statistical significance of cross-correlation values.
(1.17 MB EPS)

Figure S4 Figure S4. Time-shifted cross-correlations for the original and modified data obtained from the same cell as in Figure 5. The blue line represents the time-shifted correlations of the original EET profile and the red line represents the EET profile for the modified data, in which the large area differences for the segments in the wide protruding region between 6 and 16 min (Figure 5) are replaced with the mean area difference value of all the segments in each time frame. Similar to the red line, the green and magenta lines represent the time-shifted correlations when the retraction area (12–18 min) and both of the protrusion and the retraction areas are replaced by averaged areas, respectively. The abscissa denotes the time-shift value (plus: Rac1 activity precedes to the area differences, minus: the area difference precedes to Rac1 activity).
(0.96 MB EPS)

Figure S5 Validation of EET by permutation and rotation. The time-shifted correlation was calculated when the locations of the identified segments were rotated about the cell boundary (A) and randomly permutated (B). Each circle schematically denotes the segments we identified, and the cell edge boundary is represented by the entire linked circle. (C) The time-shifted correlations reveal that the correlation coefficients decrease with increasing rotation (blue to green lines). Red and pink lines indicate absence of correlation for permutated segments and an inverse correlation for halfway-rotated segments, respectively.
(1.10 MB EPS)

Figure S6 Comparison of morphodynamic maps and activity maps. (A) Local area difference map of a motile HT1080 cell, acquired by EET (same figure as Figure 5D). (B) Local Rac1 activity map acquired by EET on the same cell (same figure as Figure 5F). (C) Local edge movement map of the same cell by polar coordinate-based analysis. The changing color denotes temporal displacement of the cellular edge in the radial direction from the center of the cell. In contrast with the area difference map obtained by EET, the element number in each column (i.e., at each time) is consistent. The vertical and horizontal axes denote the number of degrees along the polar coordinate and the time (frame number), respectively. (D) Local Rac1 activity map by polar coordinate-based analysis on the same cell. (E) Local edge movement map of the same cell by marker-tracking-based analysis. The changing color denotes temporal perpendicular displacement of the cellular edge indicated by virtually defined markers. The vertical and horizontal axes denote the number of markers along the cellular edge and the time (frame number), respectively. (F) Local Rac1 activity map by marker-tracking-based analysis on the same cell. The maps obtained by the polar coordinate-based and the marker-tracking-based methods resemble that generated by EET.
(2.94 MB EPS)

Figure S7 Original time-shifted correlations of EET and reversely calculated time-shifted correlations. To examine the effects of the graph structure on time-shifted correlations, we obtained differently calculated time-shifted cross-correlations for the cell depicted in Figures 5, 8B, and 9C. The red line shows reversely calculated cross-correlations between each area difference and the average activities of the ancestors, while the blue line shows the original cross-correlations between each activity and the summation of the area differences of the ancestors. Note that the graph structure is the same between the two procedures. This result suggests that possible biases in the cross-correlation analysis are symmetric between positive and negative time-shifts on the graph structure obtained by EET. Although the reverse calculation with EET shows lower correlations compared to the original one, this is due to the averaging process for the activities. The averaged activities dilute the causality between segments in different time-frames because the segments are obtained based on the morphological property.
(0.96 MB EPS)

Table S1 P-values of permutation tests for two-sided test.
(0.03 MB DOC)
