# Supplementary material for: Quantification of Local Morphodynamics and Local GTPase Activity by Edge Evolution Tracking
Source: PLoS Comput Biol. 2008 Nov 14;4(11):e1000223. doi: 10.1371/journal.pcbi.1000223 (PMC2573959; doi:10.1371/journal.pcbi.1000223)
Supplement: Table S1 — P-values of permutation tests for two-sided test. (0.03 MB DOC) [file pcbi.1000223.s008.doc]

**Table S1. *P*-values of permutation tests for two-sided test.**

|  | **‑6 to 0 min** | **+6 to 0 min** | **‑6 to +6 min** |
| --- | --- | --- | --- |
| **EET-Cdc42** | 0^*^ | 0.1718 | 0.0468 |
| **EET-Rac** | 0^*^ | 0.0312 | 0^*^ |
| **EET-Rho** | 0.0938 | 0.7812 | 0.0624 |
| **Polar-Cdc42** | 0.0624 | 0^*^ | 0^*^ |
| **Polar-Rac** | 0.0312 | 0^*^ | 0.0312 |
| **Polar-Rho** | 0.0624 | 0^*^ | 0^*^ |
| **Marker-Cdc42** | 0^*^ | 0^*^ | 0.0624 |
| **Marker-Rac** | 0.2188 | 0^*^ | 0^*^ |
| **Marker-Rho** | 0.5624 | 0.1250 | 0.1250 |

*0 denotes that none of the mean values of permutated samples were greater than the original mean correlation.
